# U.S. EPA Authority to Use Cumulative Risk Assessments in Environmental Decision-Making

**DOI:** 10.3390/ijerph9061997

**Published:** 2012-05-25

**Authors:** Sarah Alves, Joan Tilghman, Arlene Rosenbaum, Devon C. Payne-Sturges

**Affiliations:** 1 ICF International, 9300 Lee Highway, Fairfax, VA 22031, USA; 2 ICF International, 2222 East NC-54, Beta Building, Suite 480, Durham, NC 27713, USA; Email: JTilghman@icfi.com; 3 ICF International, 4464 Hillview Way, Rohnert Park, CA 94928, USA; Email: ARosenbaum@icfi.com; 4 National Center for Environmental Research, U.S. Environmental Protection Agency, 1200 Pennsylvania Avenue NW, Mail Code 8723P, Washington, DC 20460, USA; Email: payne-sturges.devon@epa.gov

**Keywords:** cumulative effects, cumulative risk, environmental justice, U.S. Environmental Protection Agency, statutory authority

## Abstract

Conventionally, in its decision-making, the U.S. EPA has evaluated the effects and risks associated with a single pollutant in a single exposure medium. In reality, people are exposed to mixtures of pollutants or to the same pollutant through a variety of media, including the air, water, and food. It is now more recognized than before that environmental exposure to pollutants occurs via multiple exposure routes and pathways, including inhalation, ingestion, and dermal absorption. Moreover, chemical, biologic, radiologic, physical, and psychologic stressors are all acknowledged as affecting human health. Although many EPA offices attempt to consider cumulative risk assessment and cumulative effects in various ways, there is no Agency-wide policy for considering these risks and the effects of exposure to these risks when making environmental decisions. This article examines how U.S. courts might assess EPA’s general authority and discretion to use cumulative risk assessment as the basis for developing data in support of environmental decision-making, and how courts might assess the validity of a cumulative risk assessment methodology itself.

## List of Abbreviations

APA: Administrative Procedure Act
CAA: Clean Air Act
CEQ: Council on Environmental Quality
Cir.: Circuit
D.C. Cir.: U.S. Court of Appeals for the District of Columbia Circuit
EIS: Environmental Impact Statement
EJ: Environmental Justice
EO: Executive Order
EPA: U.S. Environmental Protection Agency
FDA: Food and Drug Administration
FDCA: Federal Food, Drug, and Cosmetic Act
FR: Federal Register
NAAQS: National Ambient Air Quality Standard
NEJAC: National Environmental Justice Advisory Council
NEPA: National Environmental Policy Act
NRC: National Research Council

## 1. Introduction

### 1.1. The Movement toward Consideration of Cumulative Risks

Historically, in its decision-making, the U.S. Environmental Protection Agency (EPA) has evaluated the risks and effects associated with exposure to a single pollutant in a single exposure medium. However, it is now more recognized than before that exposure to environmental pollutants occurs via multiple routes and pathways, including inhalation, ingestion, and dermal absorption [1,2]. Consequently, to arrive at a realistic assessment of exposure risks, regulatory authorities arguably should consider cumulative stressors [3,4,5,6] and exposure data derived from cumulative risk assessment [3,7,8].

Although many EPA offices attempt to consider cumulative effects [9,10] in various ways, there is no current Agency-wide policy to use a cumulative risk methodology to discern these effects when making environmental decisions. Some EPA offices make decisions as if they do not have the authority to use cumulative risk assessments. These office staff may believe that their authorizing statute, program implementation policies, or regulations specifically prohibit them from doing so. Further, although U.S. courts generally have accepted EPA’s authority under various statutes to use risk assessment as an analytic tool in decision-making, ([11], pp. 369–373; [12], p. 28), there has been little judicial review of the Agency’s authority to consider the results of decisions based on cumulative risk assessment. EPA’s reluctance to assert its authority in this regard suggests that the Agency may be uncertain whether a court would view statutory language directing regulation of a single category of pollutant or medium as sufficient authority to support the practice of regulating that pollutant or medium while considering the impact of multiple pollutants in various media, or considering non-environmental factors. The lack of a definitive judicial statement on whether a cumulative risk approach is permissible leaves the question open for examination. As this article will show, many environmental statutes give EPA a broad mandate (e.g., protecting public health), where there is room for interpreting the statute as allowing use of cumulative risk assessment in decision-making, even if there are few instances where the Agency has followed this approach in the past.

Box 1. **Defining Cumulative Effects.**

**DEFINING CUMULATIVE EFFECTS.**

In this article, “cumulative effects” means the qualitative and quantitative impacts from exposure to multiple chemical and non-chemical stressors–including the effects on the ecological environment, on human health, or both. This broad term includes (but is not limited to) cumulative exposures, cumulative risks, and measurable cumulative impacts. In this article, we chose cumulative risk assessment methodology as the paradigm for our analysis because it provides a concrete example of a methodology with which to discuss hypothetical court review of an EPA decision.

Further, there are compelling reasons why EPA might want to use cumulative risk assessment and the probable cumulative effects evinced through employing that methodology when making human health and ecological policy and rulemaking decisions. First, using cumulative risk assessment methodologies would present the Agency with a more accurate picture of the ecological and human health effects of its decisions than does an analysis of exposure to a single chemical or through a single medium [4,7]. Second, in 2009, the National Research Council (NRC) opined that unless EPA takes account of cumulative risks, risk assessment itself might become irrelevant in many decision contexts [13]. Third, cumulative risk assessment could be particularly helpful in addressing environmental justice (EJ) concern [14], because numerous studies have shown that minority, low-income, and indigenous communities are impacted by multiple environmental hazards, such as industrial facilities, landfills, transportation-related air pollution, poor housing, leaking underground tanks, pesticides, and incompatible land uses [15,16,17,18,19,20,21,22,23,24,25,26,27,28,29,30,31,32,33]. There is some research and analysis supporting EPA’s legal authority to consider EJ concerns under various Agency statutory authorities [34,35,36]. However, there is scarce legal research on EPA’s authority to consider cumulative effects in decision-making outside of the EJ context. 

One analysis that touches on the topic is a recently issued EPA document, *Plan EJ 2014: Legal Tools* (hereinafter referred to as *EJ Legal Tools*), which provides an overview of several discretionary legal authorities that are or may be available to EPA to address environmental justice considerations under Federal statutes and programs [36]. However, this document does not address assessing cumulative effects to develop a realistic picture of how they affect human health or the environment outside of the environmental justice context.

Analyzing cumulative effects from multiple stressors allows a more realistic evaluation of a population’s risk to pollutant exposure [15]. In a conventional risk assessment, failure to account for cumulative exposures from pollutants likely results in underestimating the combined exposure effects, to the extent that such exposures are experienced ([11], p. 362; [37], p. 117). Without an assessment of cumulative risks and probable cumulative effects, a regulatory authority charged with protecting human health and the environment may be unable to fulfill its mandate. 

### 1.2. How EPA Uses Risk Assessment

#### 1.2.1. What is Risk Assessment?

At its most general, quantified risk assessment entails the evaluation of scientific information on the hazardous properties of environmental agents, the extent of human exposure to those agents, and the risks of adverse effects (human health, or ecological effects) associated with the exposure. The product of the evaluation is a statement regarding the probability, expressed quantitatively or qualitatively, that populations so exposed will be harmed, and to what degree ([38], p. 26; [11], p. 254). Essentially, risk assessment is the process that leads to a characterization of risk ([13], p. 15). “EPA considers risk to be the chance of harmful effects to human health or to ecological systems resulting from exposure to an environmental stressor” [39].

An EPA risk assessment typically follows four basic steps:

(1) Hazard Identification: First, the Agency examines whether a stressor has the potential to cause harm to humans and/or ecological systems, and if so, under what circumstances.
(2) Dose-Response Assessment: EPA then examines the numerical relationship between exposures and effects.
(3) Exposure Assessment: The Agency then examines what is known about the frequency, timing, and levels of contact with a stressor.
(4) Risk Characterization: Finally, EPA summarizes and integrates information from the proceeding steps of the risk assessment to synthesize an overall conclusion about the nature and presence or absence of risk [40,41,42]

Cumulative risk assessment is a type of risk assessment that the NRC defines as “analysis, characterization, and possible quantification of the combined risks to health or the environment posed by multiple agents or stressors” ([13], p. 213 citing [3]). According to NRC, these stressors may be chemical, biologic, radiologic, physical, or psychologic ([13], p. 213 citing [7]). Further, these stressors may be included as quantitative or qualitative elements of an analysis, based on the complexity and context of the decision at issue ([13], p. 215). Thus, a risk assessment may be cumulative even if it lacks a quantitative analysis of all relevant non-chemical stressors ([13], pp. 217–219). For example, EPA’s Office of Pesticide Programs and Office of Solid Waste and Emergency Response conduct cumulative risk assessments to aid in decisions about pesticide regulation and Superfund sites, respectively. However, these offices’ cumulative risk assessments generally do not involve considerations of non-chemical stressors.

Because risk assessments are based on methodologies that necessarily involve a series of assumptions, which are estimated to best reflect the understanding of real-world conditions, the analysis will inevitably contain some amount of uncertainty. On its website, EPA states, “In the ideal world, all risk assessments would be based on a very strong knowledge base (*i.e.*, reliable and complete data on the nature and extent of contamination, fate and transport processes, the magnitude and frequency of human and ecological exposure, and the inherent toxicity of all of the chemicals). However, in real life, information is usually limited on one or more of these key data needed for risk assessment calculations. Consequently, risk assessors often have to make estimates and use judgment when performing risk calculations, meaning that all risk estimates are uncertain to some degree. For this reason, a key part of all good risk assessments is a fair and open presentation of the uncertainties in the calculations and a characterization of how reliable (or how unreliable) the resulting risk estimates really are” [39].

#### 1.2.2. How EPA Uses Risk Assessment in Decision-Making

EPA often supports or justifies its decision-making by estimating risks associated with various pollutants or stressors. EPA uses risk assessment to characterize the nature and magnitude of health risks to humans and ecological receptors (e.g., birds, fish, wildlife) from chemical pollutants and other stressors that may be present in the environment [39]. Typically, EPA uses risk assessments in setting health-based standards for ambient levels of environmental contaminants (e.g., National Ambient Air Quality Standards (NAAQS)), in the registration of pesticides, in permitting the manufacture of new products that use toxic substances, and in governing the reduction or concentration levels of such substances. EPA also uses these assessments in siting new polluting facilities such as waste disposal and hazardous waste facilities, in guiding cleanup levels for sites contaminated by hazardous substances, and in evaluating brownfield sites (abandoned or underutilized commercial and industrial properties). More generally, EPA may use a risk assessment whenever an environmental impact statement or environmental permit is required, in prioritizing public health concerns, and in setting priorities for research and funding ([13], p. ix; [11], p. 342).

EPA policy and decision-makers use the information developed through these assessments to help decide how to protect humans and the environment from stressors or pollutants. Although the value and relevance of risk assessments have been questioned, the NRC asserts that risk assessment remains an appropriate method for measuring the relative benefits of the many possible interventions available to improve human health ([13], p. 15).

Generally, EPA uses risk assessments that calculate expected health effects in three different statutory approaches to environmental protection: health-based provisions that typically do not tolerate “any” significant risk to public health or welfare; technology-based provisions that do not tolerate risks that can be “feasibly” eliminated; and risk-benefit provisions that find intolerable technologies, substances, or processes that pose “unreasonable” risk [43]. 

### 1.3. What this Article is About

This article explains what EPA must demonstrate to survive a legal challenge to a decision based on the results of a cumulative risk assessment. In short, the Agency must show that it has statutory authority to use such a methodology, and present evidence to support an argument that the Agency has acted rationally in the exercise of that authority. This article examines how a court would determine whether there is a defensible case that EPA has the statutory authority to use cumulative risk assessment and its products in environmental decision-making, and what evidence might be sufficient to show the rational exercise of that authority. Research suggests that the legal viability of using cumulative risk assessment to project cumulative effects depends upon the specific statutory authority under which EPA is acting, and the scientific soundness of the analysis at issue. In the context of judicial review in the United States, this inquiry is highly fact-driven and hinges on the reasonableness of the conclusions drawn from the technical analyses. 

As noted above, the analysis in this article focuses on how a court would review a challenge to EPA’s consideration of cumulative effects by basing an environmental decision on the results of a cumulative risk assessment. The reader should note that the analysis presented here is equally applicable to any construct of cumulative effects EPA might use. A reviewing court would still examine the specific statutory language at issue, the data used by EPA, and the Agency’s application or use of such data in a specific factual circumstance. Although this article presents what we believe are legally permissible interpretations of EPA authority, we acknowledge that using cumulative risk assessment as we suggest could involve legal and policy interpretations that veer from longstanding interpretations of the Agency’s program legislation and regulations. We acknowledge further that a broad use of cumulative risk assessment as the basis for EPA decision-making will require considering a mix of scientific, political, financial, human resource, and other factors. We hope that the discussion in this article can serve the continuing dialogue on this topic and advance the real-world analysis of using cumulative risk assessment in EPA policy and regulatory activity.

### 1.4. The Context for Judicial Review of the Issues in the United States

Under the Administrative Procedure Act (APA), persons aggrieved or adversely affected by agency actions, including agency promulgation of rules, have the right to seek judicial review of those actions ([44]; [45], p. 7). Most statutes establishing regulatory programs also provide for court review of agency rules ([45], p. 7). It is in this context of challenging EPA action in a U.S. court that a stakeholder might argue the Agency lacked statutory authority to consider some criteria or analytical method that EPA used in making its decision (e.g., a cumulative risk assessment).

Some environmental statutes explicitly state that human health or environmental risks must be considered, while other statutes give EPA less specific directives to protect the public health [46]. Specific legislative references to risk supply clear authority for EPA to use risk assessments in administrative decision-making. In other statutes, however, EPA has decided to key regulatory choice to level of risk without an explicit statutory mandate to do so. For example, Congress may broadly direct EPA to do what is necessary “to protect the public health;” or to address a condition that is “unsafe,” that may pose “substantial” or “significant” threat to health or safety, or that rises above a “de minimis” level of danger. Agencies often construe statutory safety or health thresholds as levels of risk [47]. 

EPA’s risk assessment emphasis is shifting from a narrow focus on single stressors, endpoints, sources, pathways, and environmental media to a broad focus on multiples of these factors [3,7,15] , resulting in a continuing, if uneven, transition to the use of cumulative risk assessment as a methodology to determine probable cumulative effects [7]. EPA has publicly embraced and encouraged the transition in Administrator announcements dating back to 1995 ([7], quoting various EPA announcements), and in the 2010 *Interim Guidance on Considering Environmental Justice During the Development of an Action*, issued by the EPA Administrator (head of the Agency) [15]. Addressed to EPA program managers, the Interim Guidance states that managers should consider cumulative effects in Agency action that may affect EJ populations [15]. However, because EPA has often faced legal challenges alleging that the Agency is over-reaching, EPA program offices may hesitate to apply a broad interpretation of the Agency’s public health statutory authority.

When presented with a challenge to its use of cumulative risk assessment in determining the probability and extent of exposure to pollutants, how might EPA persuade a court that broad or unspecific statutory language gives the Agency this authority? What might a challenger assert as a counter-argument to EPA’s claim of authority? How might a court evaluate whether a reinterpretation of authority to permit the use of a cumulative risk methodology was valid? If the Agency overcame a challenge to its authority, what evidence must it offer that a decision based on data derived from a cumulative risk assessment was rational? Sections 2.1 through 2.3 address the first three questions. Section 3 addresses the remaining question.

## 2. How a Court would Examine EPA’s Assertion of Statutory Authority to Use Cumulative Risk Assessment Methodologies in Decision-Making [48]

### 2.1. How Might EPA Persuade a Court that Broad or Unspecific Statutory Language Gives the Agency Authority to Use Cumulative Risk Assessment in Decision-Making?

Because environmental statutes may present broad mandates to protect public health or the environment, which EPA must interpret in its implementation, the question arises how reviewing courts might examine an EPA assertion that it has authority under such a mandate to use cumulative risk assessment and to act on data derived from that analysis. 

**Figure 1:**
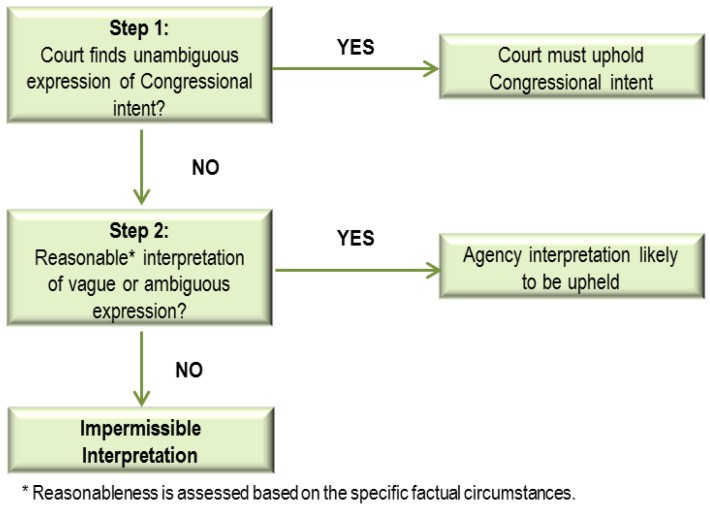
*Chevron* test—Two-part framework for resolving agency interpretations of statutes.

In the United States, when courts review an agency’s interpretation of a statute it implements, they analyze the issue under the framework laid out in the 1984 Supreme Court decision, *Chevron U.S.A., Inc. vs. Natural Resources Defense Council (Chevron)* [49]*.* In *Chevron*, the Supreme Court created a two-part framework for reviewing agency interpretations in such circumstances (Figure 1). First, a reviewing court must examine statutory language to decide whether Congress has directly spoken to the precise question at issue. If the statute is clear, the court “must give effect to the unambiguously expressed intent of Congress”, and strike down any conflicting agency interpretation ([49], pp. 842–843). However, if the relevant statutory terms do not unambiguously resolve the issue, courts must defer to the agency’s interpretation as long as it is a “reasonable” one. In specific cases of agencies acting where their authorizing statute is vague or ambiguous, a court’s analysis of reasonableness can involve complex inquiries into the specific factual circumstances of the decision, the placement of language in the relevant statute, and the legislative intent of Congress. Thus, the outcome of questions of statutory authority in cases of statutory vagueness or ambiguity turns on the specific factual circumstances surrounding a particular decision. 

*Chevron* established that courts should defer to reasonable agency interpretations of the agency’s authorizing statute where the statute is vague or ambiguous [49]. The Court reasoned, “When a challenge to an agency construction of a statutory provision, fairly conceptualized, really centers on the wisdom of the agency’s policy, rather than whether it is a reasonable choice within a gap left open by Congress, the challenge must fail” ([49], p. 866). Under the first step of the *Chevron* inquiry (*Chevron* Step One), the court independently analyzes the relevant statute to determine whether its meaning is clear. In other words, the court determines whether Congress has unambiguously either banned or required what the agency proposes to do ([50], p. 1667; citing [49], p. 842). 

Note that a court may find an unambiguous Congressional intent outside the plain words of the relevant statute. For example, in *FDA vs. Brown & Williamson Tobacco (FDA vs. Brown)*, the Supreme Court rejected a Food and Drug Administration (FDA) assertion that the agency had authority to regulate tobacco products under the Food, Drug, and Cosmetic Act (FDCA), reasoning that authority to regulate those products under the statute would require banning them from the market, based on FDA’s findings regarding the significant health risks posed by tobacco products ([51], pp. 160–161). The Court concluded that such a ban would contradict Congress’s clear intent as expressed in then-recent tobacco-specific legislation allowing the continuing sale of tobacco products in the United States ([51], pp. 137–139)*.* The court gave this rationale. “[T]he words of a statute must be read in their context and with a view to their place in the overall statutory scheme. … [T]he meaning of one statute may be affected by other Acts, particularly where Congress has spoken subsequently and more specifically to the topic at hand ([51], p. 132, citations omitted). 

If a court does find a statute ambiguous under *Chevron* Step One, it must defer to any reasonable agency interpretation under the second prong of the *Chevron* inquiry (*Chevron* Step Two). In *Chevron*, the Supreme Court explained that the power of an administrative agency to administer a Congressionally created program “necessarily requires a formulation of policy and the making of rules to fill any gap left, implicitly or explicitly, by Congress” ([49], p. 843, citing [52], p. 231). In fact, the “agency’s interpretation need not be the only permissible reading of the statute, nor the interpretation that the court might have originally given the statute” ([53], p. 581, citing [49], p. 843). 

*Chevron* suggests that if an agency interprets its enabling statute to permit making a regulatory decision rationally based on some factor or analysis that the applicable Federal statute did not specifically prohibit the agency from considering, a court would not overturn the decision simply because of reliance on the unstated factor or analysis ([50], pp. 1667–1668, 1676–1678; [54,55]). In particular, courts have made clear that agencies may consider substitute risks of proposed regulations (*i.e.*, “risk-risk” or “health-health” tradeoffs that arise when regulation of one health problem gives rise to another health problem), even where the statute the agency is implementing does not direct the agency to consider tradeoffs ([50], pp. 1672–1674, citing [56,57,58]). Courts have also often found it permissible for agencies to consider cost factors in promulgating decisions where the statute does not mention cost or feasibility ([50], pp. 1676–1678, citing [59,60,61]). Although EPA use of cumulative risk assessment is a different issue, the point here is that courts have repeatedly found agency authority to implement a statute by considering criteria not mentioned in that statute.

Under the rubric of *Chevron*, then, a court might uphold EPA’s assertion of authority to make a decision based on the results of a cumulative risk assessment even where a statute is vague, ambiguous, or silent on the subject. Section 2.2 addresses arguments that a stakeholder challenging such an EPA interpretation of its statutory authority would likely raise. 

### 2.2. What Might a Challenger Assert as a Counter-Argument to EPA’s Claim of Authority?

A stakeholder challenging EPA’s interpretation of the Agency’s statutory authority to consider the results of a cumulative risk assessment might argue that such a statutory interpretation is impermissible because the overall legislative structure for protecting human health and environmental resources prohibits considering multiple stressors outside of the pollutants addressed by a specific statute. In other words, Congress has addressed environmental pollutants and problems on a piecemeal basis, or pollutant-by-pollutant. Therefore, the argument would continue, when establishing standards for protecting the nation’s waters, for example, Congress did not intend a statute that addresses water pollutants to provide EPA with authority to consider the combined health impacts of air pollutants and water pollutants. 

A challenger making this argument would likely cite *FDA vs. Brown* (quoted above), where the Supreme Court refused to defer to a Federal agency’s interpretation of its statutory authority, finding that the result of such agency interpretation would be inconsistent with other Congressional and agency action [51]. Applying the Supreme Court’s reasoning in *FDA vs. Brown*, one could argue that because Congress addressed different pollutants and the protection of different media under different statutory schemes, it did not intend for EPA to consider the cumulative effects of all pollutants when determining how stringently to limit one type of pollutant.

On the other hand, in *Massachusetts vs. EPA*, the Supreme Court found that EPA could find carbon dioxide to be an air pollutant under the Clean Air Act based on that pollutant’s contribution to global climate change, although EPA argued the Clean Air Act did not contemplate the regulation of substances that contribute to climate change ([62], p. 528). In many environmental statutes, Congress has given EPA undefined directives to do what is necessary to protect the public health, presumably to allow the statute to remain flexible over time so that the Agency could decide whether to restrict pollutants based on the latest scientific advancements affecting the EPA’s understanding of health effects ([62], p. 532). Indeed, in *Massachusetts vs. EPA*, the Supreme Court explained that although “the Congresses that drafted the [1970 Clean Air Act] might not have appreciated the possibility that burning fossil fuels could lead to global warming, they did understand that without regulatory flexibility, changing circumstances and scientific developments would soon render the Clean Air Act obsolete” ([62], p. 532). Therefore, although one could argue that a court should view the Federal environmental regulatory structure as forbidding EPA to consider cumulative effects of multiple pollutants under a statute that addressed only one medium or type of pollutant, one reading of *Massachusetts vs. EPA* is that Congress purposefully writes statutes broadly so that EPA can implement them more effectively over time. This reading lends support to the argument that EPA could interpret a broad statutory mandate to protect the public health as permitting it to consider cumulative risks, if the Agency reasonably determined that the consideration of such evidence was necessary to effectively carry out its statutory mandate ([62], pp. 529–532). 

A challenger also might cite *North Carolina vs. EPA*, to support an assertion that EPA was without authority to consider the results of a cumulative risk assessment [63]. In that case, the D.C. Circuit rejected EPA’s interpretations of its Clean Air Act authority, finding that the Agency went beyond its statutory authority when it established a regional interstate emissions trading program in an attempt to streamline separate Clean Air Act requirements ([63], pp. 907–922). Some might argue that the court’s analysis in *North Carolina vs. EPA* signals the D.C. Circuit’s hesitance to permit broad interpretations of the Clean Air Act and other statutes because the court interpreted the provision at issue so narrowly [64]. Others might argue that the *North Carolina vs. EPA* case implies the court’s willingness to check what might be seen as excessive EPA action, and to require Congressional action to significantly change existing programs. However, the EPA statutory interpretation under review in *North Carolina vs. EPA* was quite different from the statutory interpretations suggested in this article.

In that case, the court vacated an EPA rule for many reasons, but most notably for this discussion, because the statutory provision at issue provided a very specific instruction rather than the broad statutory mandates to protect the public health we are addressing in this article. In pertinent part, the provision at issue said that each State Implementation Plan must ensure the prevention of “any…type of emissions activity” that “contributes significantly to nonattainment in, or interferes with maintenance by, any other State with respect to any [NAAQS]” [65]. This specific instruction contrasts with the broad statutory mandates to protect the public health we are addressing in this article, e.g., to establish standards “requisite to protect the public health” while “allowing an adequate margin of safety” (the NAAQS standard setting authority) [66]. Because these two grants of authority are distinguishable, *North Carolina vs. EPA* does not preclude the D.C. Circuit from upholding EPA’s interpretation of its authority to consider any information or analyses the Agency reasonably determines is necessary to decide the level at which standards are protective of the public health.

In *North Carolina vs. EPA*, the D.C. Circuit also found that the rule at issue conflicted with the Clean Air Act provision that established a compliance deadline for certain NAAQS. Therefore, in *North Carolina vs. EPA*, the Agency was interpreting its statute in such a way that conflicted with another provision of the statute ([63], pp. 911–912). In contrast, the analysis in this article is suggesting that EPA could interpret broad authority to protect the public health to include a consideration of background effects from multiple stressors that might affect how the pollutant at issue impacts human health or the environment. This analysis does not suggest that EPA could interpret its statutory directive to conflict with another provision of the statute. In fact, as discussed in greater detail below, EPA must assess all criteria that Congress has directed the Agency to consider in making its determinations under a particular statute ([67], p. 43). If EPA concludes that basing a decision on results from a cumulative risk assessment is the best way to carry out a statutory mandate to protect the public health, and does so in a way that is not in conflict with any other provisions of the statute, a court likely would defer to the Agency’s reasonable interpretation of its statutory authority ([49], p. 843).

For these reasons, the authors conclude that *North Carolina vs. EPA* may be consistent with the D.C. Circuit’s application of *Chevron*’s principle of deference to reasonable EPA interpretations of the Agency’s statutory authority. Assuming that EPA can overcome the arguments discussed in this section, there is another principle of statutory construction to consider: whether a U.S. Federal agency may change a longstanding interpretation of its authorizing legislation. Section 2.3 addresses how a court would view an EPA interpretation of its statute that differed from how the Agency had interpreted the same statute in the past.

### 2.3. How might a Court Evaluate whether a Reinterpretation of Authority to Permit the Use of a Cumulative Risk Methodology was Valid?

Some EPA offices may hesitate to use cumulative risk assessment in program decision-making where the Agency previously has not considered cumulative risks in implementing that program. Courts may question the validity of an agency’s reinterpretation of its substantive legislation [68]—especially if the earlier interpretation received court approval [69,70], or appears to be more consistent with other Congressional and agency action ([51], pp. 138–139). However, the Supreme Court has recognized that agency interpretations of their substantive authorities are not immutable, and thus may be changed when appropriate ([49], pp. 863–864). In the *Chevron* case, the Supreme Court stated, “An initial agency interpretation is not instantly carved in stone. On the contrary, the agency, to engage in informed rulemaking, must consider varying interpretations and the wisdom of its policy on a continuing basis” ([49], pp. 863–864). 

Therefore, courts’ deference to reasonable EPA interpretations under *Chevron* Step Two (Figure 1) extends not only to EPA’s initially selected interpretation, but also to subsequent decisions to change its preferred interpretation ([71], p. 742; [72], p. 521; [73], pp. 22–27; [74] p. 317; [75]). In such cases, the court would apply the presumption that when Congress left an ambiguity in a statute meant for implementation by an agency, Congress understood that the implementing agency—not the reviewing court—would have discretion to resolve the ambiguity ([71] citing [49]). Based on a review of the case law, Table 1 presents some of the factors a court might consider when determining whether an agency has authority to changes its interpretation of a statute that agency implements.

**Table 1:** Factors a court might consider when determining whether an agency has authority to apply a new interpretation of its authorizing legislation.

Can an agency change its interpretation of authorizing legislation?	
Unlikely if…	Likely if…
• Old interpretation has received court approval;	• The agency provides a rationale for the change;
**OR**	**AND**
• Old interpretation is consistent with other Congressional or agency action;	• New evidence supports a different interpretation to satisfy the statutory mandate;
**OR**	**AND**
• New interpretation is arbitrary, capricious, or an abuse of discretion.	• The agency provides adequate notice of and opportunity to comment on methodology change.

It would seem, then, that under the *Chevron* test, a court might uphold an EPA interpretation that a statutory mandate to “protect the public health” may authorize the use of a cumulative risk assessment methodology, even if this determination overturns a longstanding interpretation of the underlying legislation. Such a result seems most likely where the Agency reasonably interpreted the statute as permitting consideration of cumulative effects derived from a cumulative risk assessment methodology, and where EPA has strong scientific evidence to support the assertion that cumulative effects are a significant concern in the circumstance at issue ([11], p. 378). However, assuming that a court would uphold EPA’s interpretation or reinterpretation of a vague or ambiguous statute to permit the use of cumulative risk assessment, there remains a further legal hurdle. A challenger still could assert that even assuming the Agency had authority to use the methodology and results of the analysis, there were flaws in the conduct of the analysis itself or in the use of the results. In such a case, EPA must show that there was a “rational basis” for its decision–that its actions were not “arbitrary and capricious.” A U.S. court would analyze these issues under the rubric of the APA [44].

## 3. If EPA Overcame a Challenge to Its Authority, What Evidence must It Offer that a Decision Based on Data Derived From a Cumulative Risk Assessment was Rational?

As noted above, EPA often supports or justifies its decision-making by estimating risks associated with various pollutants or stressors. This activity usually results in an administrative rule setting a particular health, ecological, or technology-based standard or policy. Assuming a court has found that EPA had authority to consider cumulative risk in an ambiguous statutory directive, how might a court assess whether there is a rational basis for using a cumulative risk assessment methodology and the results deriving from it? This inquiry is particularly important given that the product of any risk assessment is a qualitative or quantitative statement regarding the probability of, and degree to which exposed populations or systems will be harmed ([38], p. 26; [11], p. 354). In other words, how does EPA survive the “arbitrary and capricious” test when it employs a methodology that necessarily involve a series of assumptions, which are estimated to best reflect the Agency’s understanding of real-world conditions, and inevitably contain some amount of uncertainty ([13], p. 19)?

**Figure 2:**
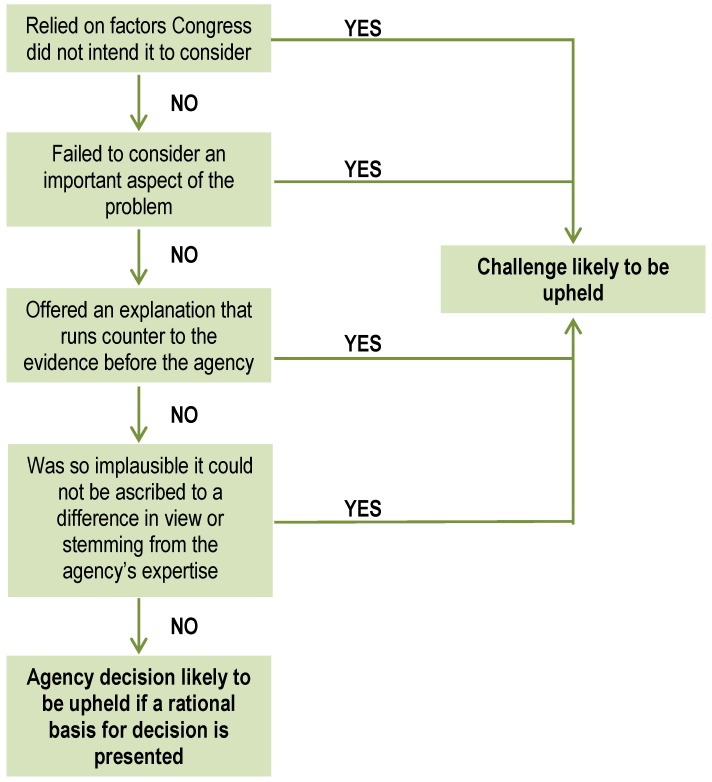
Under the APA, the court will conduct a “searching and careful” review and ask whether the agency made a decision that was not “arbitrary and capricious”. (Note that the steps are not necessarily sequential.)

In the United States, the APA provides the basic framework within which Federal agencies must operate in promulgating rules, issuing policy statements, and adjudicating rights [44]. Under this statute, a court may assess the validity of regulatory agency decision-making, and “hold unlawful and set aside agency action, findings, and conclusions found to be arbitrary, capricious, an abuse of discretion or otherwise not in accordance with law” [76]. In applying this “arbitrary and capricious” standard, a court must conduct a “searching and careful” review of the agency’s record ([67], p. 43). The court must vacate the action if “the agency has relied on factors which Congress has not intended it to consider, entirely failed to consider an important aspect of the problem, offered an explanation for its decision that runs counter to the evidence before the agency, or is so implausible that it could not be ascribed to a difference in view or the product of agency expertise” ([67], p. 43) (Figure 2). 

Although the APA requires courts to perform a “searching and careful” inquiry into the facts underlying the agency’s decisions, courts will “presume the validity of agency action as long as ‘a rational basis for it is presented’” ([56], p. 362, citing [77], p. 1145) (Figure 2). U.S. courts generally give an “extreme degree of deference to an agency that is evaluating scientific data within its technical expertise,” reviewing the agency’s action to “ensure that [the agency] has examined the relevant data and has articulated an adequate explanation for its action” ([78], p. 247, internal quotation marks omitted). Indeed, most courts will grant an agency considerable deference for its “scientific procedures as long as there has been sufficient evidence in the record and sufficient explanation for the action, even though different inferences might have been drawn from the same data and theories and even though courts themselves sometimes suggest they might have drawn different conclusions” ([11], p. 377). 

Courts have explained and illuminated the scope of judicial review in the face of administrative decision-making where, as in the case of cumulative risk assessment, the process contains inherent technical judgment and complexity. In the 2008 case, *Northwest Coalition for Alternatives to Pesticides vs. EPA*, the 9th Circuit Court of Appeals majority opinion quoted the dissenting opinion of a 1985 D.C. Circuit case: “Although the ultimate scope may be narrow, the depth must be sufficient for us to be able to comprehend the agency’s handling of the evidence cited or relied upon. The purpose of this in-depth review is to educate ourselves so that we can properly perform our reviewing function: determining whether the agency’s conclusions are rationally supported. …[W]here the agency’s reasoning, although complex, is rational, clear, and complete, we must affirm. Contrarily, where the agency’s reasoning is irrational, unclear, or not supported by the data it purports to interpret, we must disapprove the agency’s action” ([79], p. 1052 n.7).

Other recent cases provide examples of different courts acting on the principle of applying this “extreme” or “considerable” deference to Federal agency decisions in the scientific context. In *Coalition of Battery Recyclers Ass’n vs. EPA* (2010), the D.C. Circuit upheld EPA’s shift in focus from blood lead levels in the original 1978 NAAQS for lead to IQ decrements in children in the revised lead NAAQS. In support of its ruling, the court noted that EPA explained in both the proposed and final rule that current scientific evidence no longer recognized a safe blood level for lead, that epidemiological studies of cognitive effects and lead exposure commonly used IQ scores, and that the scientific literature supported the conclusion that lead exposure causes IQ loss in children ([80], pp. 618–619; [81,82,83]). 

In *Tucson Herpetological Society vs. Salazar* (2009), the 9th Circuit Court of Appeals upheld the Department of Interior’s assessment of threats to a lizard’s habitat in its decision not to list the species as threatened under the Endangered Species Act [84]. In explaining that the merits of the challengers’ and the agency’s conflicting scientific studies are not a proper subject for the court to resolve, the court cited a 2007 ruling in which it noted that courts must defer to an agency’s interpretation of complex scientific data ([84], pp. 881–882, citing [85], p. 1150). The court also cited a 1989 Supreme Court opinion explaining that when specialists express conflicting views, an agency must have discretion to rely on the reasonable opinions of its own qualified experts even if, as an original matter, a court might find contrary views more persuasive ([84], p. 882, citing [86], p. 378). 

In *Miami-Dade County vs. EPA* (2008), the 11th Circuit Court of Appeals found that EPA’s risk assessment methodology and results bore a rational relationship to the characteristics of the data to which it was applied ([87], pp. 1063–1071). The court upheld the Underground Injection Control Program rule that the risk assessment at issue supported against varying challenges that EPA’s risk assessment both underestimated and overestimated actual risks ([87], pp. 1063–1071). The court explained that where a “statute is precautionary in nature, the evidence is difficult to come by, uncertain, or conflicting because it is on the frontiers of scientific knowledge, the regulations designed to protect the public health, and the decision that of an expert administrator, we will not demand rigorous step-by-step proof of cause and effect” ([87], pp. 1064–1065, citing [12], p. 28). According to the court, because the assumptions EPA applied in its risk assessment methodology bore a rational relationship to the real world, given the incomplete information faced by the Agency, the court was obligated to uphold EPA’s technical judgment ([87], p. 1070).

In *Mossville Environmental Action Now vs. EPA* (2005), the D.C. Circuit remanded an EPA determination to use vinyl chloride as a surrogate for other hazardous air pollutants. The court reasoned that it could not assess the rationality of the Agency’s analysis, because EPA failed to memorialize evidence of the correlation the Agency claimed existed between vinyl chloride and the other pollutants in the record ([88], p. 1243). Thus, a court will find an EPA decision arbitrary and capricious if EPA fails to show a rational relationship between its conclusions or assumptions and the evidence before the Agency as contained in the record. In summary, a review of the case law indicates that when a stakeholder challenges the quality of the data or technical process relied on by EPA or suggests that other data is more persuasive, courts are likely to defer to EPA’s expertise and uphold the final agency action. In contrast, challengers tend to succeed when the record under review shows data gaps or missing steps in EPA’s logic that preclude meaningful review of EPA’s decision-making process ([74], pp. 318–319). 

Given the inherent scientific judgment in the selection of data and assumptions at various steps of a risk assessment, a court will attempt to ensure that EPA performed the most rigorous analysis possible given the available data ([87], pp. 1069–1070). If the data and assumptions upon which a cumulative risk assessment is based are such that a reasonable policy-maker could not interpret the results of the risk assessment with reasonable confidence that those results bear a rational relation to the real world (*i.e.*, if the uncertainty of the risk assessment findings is too high), a court would likely find it inappropriate to consider such health effects in agency decision-making ([79] quoting [89], p. 1373). This is because a court will overturn an agency decision where the agency fails to provide sufficient information to demonstrate a rational connection between the factors that agency examined and the conclusions it reached. For example, in *Northwest Coalition for Alternatives to Pesticides vs. EPA*, the 9th Circuit Court of Appeals rejected EPA’s choice of a safety factor as arbitrary where the court was unable to determine whether there was reliable data supporting the Agency’s choice of that factor. The court found that EPA failed to explain the connection between the toxicological data and the safety factor selected ([79], p. 1052). Therefore, if EPA does not explain how its reliance on the results of a cumulative risk assessment relates to its statutory directive, or if EPA does not explain how its decision is supported by the results, a court would likely overturn that decision.

It follows that when a challenger argues that an EPA decision is flawed due to a faulty risk assessment, to avoid a ruling that it has been arbitrary and capricious, the Agency must show a rational relationship between the assumptions used in the risk assessment and what is known from real-world data [87,90]; and that it considered all important aspects of the problem ([67], p. 43). A plausible way for EPA to show that its use of results from a cumulative risk assessment was not arbitrary and capricious is to present evidence that the cumulative risk assessment was based on reasonable methodologies and assumptions, given the available data, and that EPA’s use of the risk assessment results were reasonable based on the analysis. 

Court cases that have addressed challenges to EPA risk assessments confirm this understanding. In *Miami-Dade County vs. EPA* (discussed above), the 11th Circuit Court of Appeals upheld EPA’s use of conservative risk assessment assumptions [91,92] as a means to address uncertainties. The court found these assumptions to represent a legitimate discretionary decision-making methodology because it was rational for EPA to err on the side of overprotection when faced with data uncertainties ([87], pp. 1069–1070, citing [90,93,94]). In *West Virginia vs. EPA* (2004), the D.C. Circuit explained that deference is due to an agency’s modeling of complex phenomena, so long as “model assumptions...have a ‘rational relationship’ to the real world” ([93], pp. 866–867). In the *American Iron & Steel* case (1997), the D.C. Circuit stated that “it is within EPA’s discretion to decide that in the wake of uncertainty, it would be better to give the values a conservative bent rather than err on the other side” ([90], p. 993). The D.C. Circuit found EPA was “reasonable” in using human health uncertainty factors in a risk assessment where the factors were created as a function of the available data ([90], p. 993). In multiple decisions, courts have noted that “[T]he law does not require selection of the single best methodology in any case, but only a study based on consideration of the relevant factors and in the construction of which there has been no clear error of judgment” ([87], p. 1069, internal quotes omitted, citing [95], p. 416). 

Decision-makers should be mindful that courts often will probe deeply into the science and reason underlying an agency’s decision when a challenger asserts that a choice of methodologies or studies was unreasonable, or that the agency failed to consider a plausible alternative ([96] citing [79], p. 1052 n.7; [72,97,98,99]). Therefore, if a stakeholder challenges EPA’s use of a cumulative risk assessment methodology when making a decision to set standards “requisite to protect the public health,” the Agency must be able to show that it rationally determined from the available data that it should consider cumulative risks to implement the statutory mandate effectively ([11], p. 378). Such an interpretation of a broad public health mandate arguably would be reasonable in light of compelling scientific evidence of a cumulative adverse health effect ([84] citing [86], p. 378; [87]). One could argue that where new scientific evidence indicates a different approach from the longstanding one would better serve a statutory mandate, Federal agencies should change their standard-setting methodology [49,100]. 

## 4. Conclusions

The question of whether EPA may survive a legal challenge to its discretion to make decisions based on data derived from cumulative risk assessment rests on two questions. The first and preeminent question is whether the Agency may interpret or reinterpret its various enabling statutes as providing authority to use this methodology. Assuming EPA has such authority, the second question is whether the cumulative risk assessment methodology and the results derived from that analysis are reasonable, based on the available data. This article suggests that environmental statutes directing EPA to “protect the public health” (or some other similarly broad mandate to protect human health or the environment) likely provide authority for the Agency to use this methodology in various types of decision-making, even if the Agency reinterpreted a statute to provide this authority. 

In *Chevron*, the Supreme Court created a two-part framework for reviewing agency interpretations of a statute it implements [49]. At *Chevron* Step One, a reviewing court examines statutory language to determine whether Congress has spoken directly to the issue. Since many environmental statutes direct EPA to take actions necessary to “protect the public health” or to address a condition that may pose a “substantial” or “significant” thread to public health, a court would likely find that Congress has not directly spoken to whether EPA can consider cumulative risks in making such decisions. If a court does find a statute ambiguous under *Chevron* Step One, it must defer to any reasonable agency interpretation under *Chevron* Step Two. *Chevron* suggests that if an agency makes a regulatory decision rationally based on some factor or analysis that the applicable Federal statute did not specifically prohibit the agency from considering, a court would not overturn the decision simply because of reliance on the unstated factor or analysis.

Given the plausible argument that EPA has authority to use cumulative risk assessment to support decision-making under a statute, there remains the question of whether EPA’s use of cumulative risk assessment in a particular circumstance can withstand arbitrary or capricious review under the APA. Whether a particular cumulative risk analysis is appropriate for consideration in an Agency policy or regulatory decision is a fact-driven inquiry requiring judicial examination on a case-by-case basis. Therefore, there is no way to make a broad statement of general applicability regarding how to construct a cumulative risk assessment analysis that would be upheld in a court of law; in different factual circumstances, the sufficiency of evidence of the risks and exposure effects shown by the analysis may be different. A reviewing court’s inquiry into the reasonableness of a cumulative risk assessment likely would examine whether the data and assumptions used in the assessment were rational, based on the available information, and whether the Agency’s conclusions were reasonable based on the analysis. If data and assumptions are sufficient for a court to decide “whether the agency’s conclusions are rationally supported,” the court must affirm ([79], p. 1052 n.7).

In many current circumstances, making reasonable quantitative estimates of impacts using cumulative risk assessments would require access to data that currently are nonexistent or insufficient. Recently, potential users in the scientific community have suggested the necessity and value of having the following cumulative effects data and tools: larger emissions inventories, air quality monitoring networks, modeling software, and pollution inventories expanded to unregulated operations [101,102,103,104]. The writers believe that EPA and the scientific community should focus on developing the necessary data and tools to provide a sound cumulative risk assessment framework. The Agency then should develop guidelines for a cumulative risk assessment methodology, including guidance for evaluating qualitative cumulative effects.

To support EPA use of cumulative effects methodologies in environmental decision-making, future research could include analyses of specific EPA decisions under particular statutes. EPA acknowledges the situation-specific nature of legal challenges in its recent *EJ Legal Tools* document [36]. *EJ Legal Tools* analyzes EPA’s statutes and their relevant regulatory standards to protect public health or welfare and the environment for how they might provide opportunities to ensure that Federal policies have no disproportionately high and adverse human health or environmental effects on minority or low-income communities ([36], p. 2). The Agency explains that without the context of specific applications, the *EJ Legal Tools* document does not attempt to fully characterize the legal risks of interpreting EPA’s “more vague” legal authorities ([36], p. 2). Although we discuss EPA’s general authority in this article, future research could include a closer examination of how specific legal authorities might allow EPA discretion to consider cumulative effects, and how courts interpreted that EPA statutory provision over time.
